# Supplementary material for: Invariant representation of physical stability in the human brain
Source: eLife. 2022 May 30;11:e71736. doi: 10.7554/eLife.71736 (PMC9150889; doi:10.7554/eLife.71736)
Supplement: Supplementary file 1. — Each cell shows the average value of the variable for stable and unstable conditions along with the p value for a paired t-test comparing the two sets of values across subjects. The first five columns correspond to eye movement variables collected on 6 subjects during the fMRI experiment and the last column is for the interestingness rating collected on the same set of images but in 11 subjects outside the scanner (see Methods for details). Since the subjects were instructed to maintain fixation at the center of the image, we did not observe any saccadic events (amplitude >1°) and hence we are calling the small ballistic events as simply eye movements. Significant effects (p < 0.05) are highlighted in bold. [file elife-71736-supp1.docx]

Physical- Objects


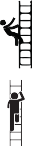
Physical- People

Animals- People

unstable stable

unstable stable

unstable stable


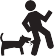

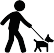


X location *(px)* Y location *(px)*

Screen resolution: 1024 x 768

(512, 384) is the fixation point

| 518.1 | 388.5 | 36.6 | 40.6 | 1.13 |
| --- | --- | --- | --- | --- |
| 505.6 | 380.6 | 29.1 | 47.9 | 1.14 |
| *p = 0.12* | *p = 0.19* | *p = 0.1* | *p = 1* | *p = 0.93* |
| 517.8 | 377.2 | 37.7 | 30.4 | **1.31** |
| 516.9 | 370.8 | 31.9 | 39.9 | **1.08** |
| *p = 0.81* | *p = 0.6* | *p = 0.13* | *p = 0.62* | ***p = 0.028*** |
| 507.6 | 388.9 | 40.8 | 57.3 | 1.38 |
| 512.1 | 379.7 | 43 | 41.1 | 1.26 |
| *p = 0.81* | *p = 0.19* | *p = 0.45* | *p = 0.31* | *p = 0.26* |

# Number of eye movements

# Duration of eye movements *(ms)*

Amplitude of eye movements *(minutes of arc)*

Interestingness rating *(scale 1-5)*

| **2.9** |
| --- |
| **1.29** |
| ***p = 0.0007*** |
| **3.69** |
| **1.97** |
| ***p < 0.0001*** |
| **3.84** |
| **2.7** |
| ***p = 0.0006*** |
