# Supplementary material for: Invariant representation of physical stability in the human brain
Source: eLife. 2022 May 30;11:e71736. doi: 10.7554/eLife.71736 (PMC9150889; doi:10.7554/eLife.71736)
Supplement: Supplementary file 2. — Each cell shows the average Fisher transformed within and between stability condition pattern correlations along with the p value for a paired t-test comparing the two sets of values. Each column includes the results from one fROI. The top three rows contain results for within-scenario pattern correlation analysis and the bottom three rows show results for the pattern correlation analysis across scenarios. Significant effects with within condition correlations greater than between condition correlations are highlighted in bold. [file elife-71736-supp2.docx]

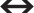


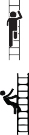

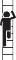

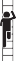

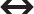
Physical- Objects


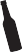


|  | # voxels: mean ± sem 273 ± 57.9 | # voxels: mean ± sem 64.2 ± 13.6 |
| --- | --- | --- |
| within | **0.72** | 0.42 |
| between | **0.58** | 0.37 |
|  | ***p = 0.011*** | *p = 0.18* |
| within | **0.66** | 0.36 |
| between | **0.57** | 0.29 |
|  | ***p = 0.002*** | *p = 0.27* |
| within | 0.54 | 0.51 |
| between | 0.59 | 0.51 |
|  | *p = 0.31* | *p = 0.88* |


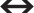


Physical- People


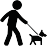

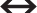

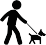


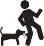

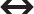

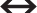

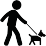
Animals- People

Parietal Physics ROI


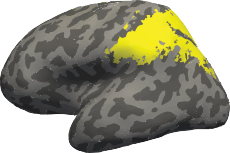


Frontal Physics ROI


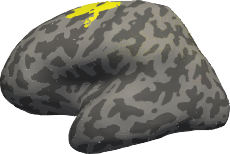


Physical- People

Animals- People

Physical- Objects


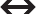


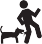

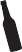

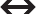

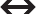

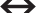

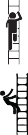

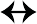

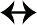

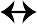
Physical- Objects


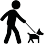

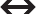


Physical- People


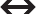


Animals- People

within between

within between


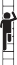

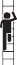


| 1.13 | 1.25 |
| --- | --- |
| 1.02 | 1.16 |
| *p = 0.077* | *p = 0.19* |
| 1.20 | 1.28 |
| 1.12 | 1.17 |
| *p = 0.18* | *p = 0.16* |
| 1.06 | 1.24 |
| 0.99 | 1.27 |
| *p = 0.37* | *p = 0.77* |


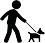

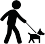
 within between
