# Supplementary material for: Invariant representation of physical stability in the human brain
Source: eLife. 2022 May 30;11:e71736. doi: 10.7554/eLife.71736 (PMC9150889; doi:10.7554/eLife.71736)

**Average beta estimates**

Physical- Objects

|  | # voxels: mean ± sem 273 ± 57.9 | # voxels: mean ± sem 64.2 ± 13.6 |
| --- | --- | --- |
| unstable | **0.16** | 1.75 |
| stable | **-0.03** | 1.8 |
|  | ***p = 0.02*** | *p = 0.43* |
| unstable | **0.21** | 1.93 |
| stable | **0.03** | 1.95 |
|  | ***p = 0.04*** | *p = 0.7* |
| unstable | 0.075 | 2.08 |
| stable | 0.095 | 2.2 |
|  | *p = 0.67* | *p = 0.09* |


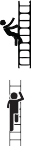
Physical- People

Animals- People


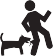

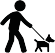


Parietal Physics ROI


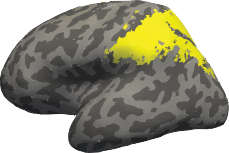


Frontal Physics ROI


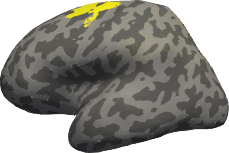

Supplement: Supplementary file 3. — Each cell shows average GLM estimated beta values for unstable and stable conditions along with the p value for a paired t-test comparing the two sets of values across subjects. Scenarios showing significantly higher response to unstable scenes compared to stable scenes are highlighted in bold in each column. [file elife-71736-supp3.docx]
