# Supplementary material for: Invariant representation of physical stability in the human brain
Source: eLife. 2022 May 30;11:e71736. doi: 10.7554/eLife.71736 (PMC9150889; doi:10.7554/eLife.71736)
Supplement: Supplementary file 4. [file elife-71736-supp4.docx]

Supplementary Table 4: Reaction times for the stability judgement task

|  | Physical-Objects | | Physical-People | | Animals-People | |
| --- | --- | --- | --- | --- | --- | --- |
|  | Stable | Unstable | Stable | Unstable | Non-Peril | Peril |
| Reaction Time, ms  (mean ± std) | 862.1 ± 43.8 | 943.2 ± 45.2 | 1088.2 ± 48.8 | 958.1 ± 46.8 | 1003.2 ± 60.8 | 881.5 ± 29.9 |
| p-value  (signrank test on avg. RTs across subjects) | p = 0.05 | | p = 0.002 | | p = 0.008 | |
| Average RT, ms | 902.7 | | 1023.2 | | 942.3 | |
| RT difference, ms  (unstable – stable) | 81.2 | | -130.1 | | -121.7 | |
