# Supplementary material for: Invariant representation of physical stability in the human brain
Source: eLife. 2022 May 30;11:e71736. doi: 10.7554/eLife.71736 (PMC9150889; doi:10.7554/eLife.71736)
Supplement: Supplementary file 5. [file elife-71736-supp5.docx]

Supplementary Table 5: Accuracy of stability judgement

|  | Physical-Objects | | Physical-People | | Animals-People | |
| --- | --- | --- | --- | --- | --- | --- |
|  | Stable | Unstable | Stable | Unstable | Non-Peril | Peril |
| % Accuracy (mean ± std across subjects) | 94.4 ± 1.9 | 93.1 ± 1.9 | 88.5 ± 3.3 | 95.7 ± 1.4 | 93.5 ± 1.4 | 99.5 ± 0.4 |
| p-value (signrank test on avg. accuracy across subjects) | p = 0.67 | | p = 0.08 | | p = 0.002 | |
