# Supplementary material for: Invariant representation of physical stability in the human brain
Source: eLife. 2022 May 30;11:e71736. doi: 10.7554/eLife.71736 (PMC9150889; doi:10.7554/eLife.71736)
Supplement: Supplementary file 6. [file elife-71736-supp6.docx]

*Stability Detection Behavioral Experiment:*

*Methods:* To test whether the images in the three scenarios systematically differed in ease of understanding, we ran a behavioral task where we asked subjects (n = 16) to categorize the stability of each image. Each trial started with the presentation of the image (along with a fixation cross) at the center of the screen for 1.8s followed by a fixation-only inter-trial interval of up to 3.2s. The participants were asked to press either ‘S’ (for stable or nonperilous) or ‘N’ (for unstable or perilous) key on a standard QWERTY keyboard to indicate the stability of the presented image as quickly and as accurately as possible. Images from all scenarios and conditions were randomly interleaved.

*Results:* We found that reaction times differed significantly between stable and unstable conditions within each scenario, but the direction of the effect was not consistent across the scenarios (see Supplementary Table 4) and – most importantly – the overall pattern of RT and accuracy is not consistent with an account of our fMRI findings in terms of difficulty. Specifically, although the Animals-People condition was easier overall than the Physical-People condition both in terms of accuracy (p = 0.047 for signrank test on avg accuracy across subjects; Supplementary Table 5) and reaction time (p = 0.004 for signrank test on avg RT across subjects), it is the *difference in difficulty between the unstable/perilous conditions versus the stable/nonperilous conditions* that is important because it is this comparison that our fMRI results are based on. And here, we find that in fact subjects were *faster* (p = 0.002) and *more accurate* (p = 0.08; tending towards significance) on the unstable than stable conditions in the Physical-People scenarios, an effect that goes in the opposite direction from the difficulty hypothesis. Further, although participants did take more time to categorize the unstable than stable stimuli in the Physical-Objects condition (p = 0.05), this effect was smaller in magnitude than that found for the Animals-People control scenario (p = 0.033 for interaction between scenario and stability in an ANOVA) where we did not find similarly higher activations for unstable/perilous than stable/nonperilous conditions. Thus, our behavioral data are not consistent with the hypothesis that any of our important fMRI findings (either univariate or multivariate) reflect difficulty confounds.
